# Supplementary material for: Complexome profiling of the Chlamydomonas psb28 mutant reveals TEF5 as an early PSII assembly factor
Source: Plant Cell. 2025 Mar 17;37(6):koaf055. doi: 10.1093/plcell/koaf055 (PMC12164590; doi:10.1093/plcell/koaf055)
Supplement: koaf055_Supplementary_Data [file koaf055_supplementary_data.zip › psb28_interactive.zip!/plots/intensity/qualityPlots/peptides/AXT2_Cre07.g332500.t1.2.html]

### Cre07.g332500.t1.2

Trivial name: AXT2  
SeqCoverage [median]: 6.10  
Mass: 81.60 kDa  
  
 

| Sequence | MissedCleavage | Mass [Da] | wtIntensity | muIntensity | Score |
| --- | --- | --- | --- | --- | --- |
| EYSFFNNPR | 0 | 1172.53 | 0.00 | 651.17 | 55.68 |
| MLQNAAAAAER | 0 | 1144.57 | 0.00 | 475.05 | 48.28 |
| SAIGVALTLGR | 0 | 1056.63 | 0.00 | 637.16 | 60.10 |
|  |  |  | 0.0 | 1763.4 |  |
